# Oxamic transcarbamylase of *Escherichia coli* is encoded by the three genes *allFGH* (formerly *fdrA, ylbE*, and *ylbF*)

**DOI:** 10.1128/aem.00957-24

**Published:** 2024-06-18

**Authors:** Nam Yeun Kim, Ok Bin Kim

**Affiliations:** 1 Division of EcoScience, Department of Life Science, Ewha Womans University, Seoul, Republic of Korea; University of Milano-Bicocca, Milan, Italy

**Keywords:** *allFGH*, oxamic transcarbamylase, carbamoyl phosphate, global orphan enzyme, allantoin degradation

## Abstract

*Escherichia coli* uses allantoin as the sole nitrogen source during anaerobic growth. In the final step of allantoin degradation, oxamic transcarbamylase (OXTCase) converts oxalurate to carbamoyl phosphate (CP) and oxamate. The activity of this enzyme was first measured in *Streptococcus allantoicus* in the 1960s, but no OXTCase enzyme or the encoding gene(s) have been found in any strain. This study discovered that *allFGH* (*fdrA*, *ylbE*, and *ylbF*) are the genes that encode the global orphan enzyme OXTCase. The three genes form an operon together with *allK* (*ybcF*), encoding catabolic carbamate kinase. The *allFGHK* operon is located directly downstream of the *allECD* operon that encodes enzymes for the preceding steps of OXTCase. The OXTCase kinetic parameters were analyzed using the purified protein composed of AllF-AllG-AllH (FdrA-YlbE-YlbF); for the substrate CP, *K*_*M*_ and *V*_max_ were 1.3 mM and 15.4 U/mg OXTCase, respectively, and for the substrate oxamate, they were 36.9 mM and 27.0 U/mg OXTCase. In addition, the OXTCase encoded by the three genes is a novel transcarbamylase that shows no similarity with known enzymes of the transcarbamylase family such as aspartate transcarbamylase, ornithine transcarbamylase, and YgeW transcarbamylase. The present study elucidated the anaerobic allantoin degradation pathway of *E. coli*. Therefore, we suggest that the genes *fdrA*, *ylbE*, and *ylbF* are renamed *allF*, *allG*, and *allH*, respectively.

**IMPORTANCE:**

The anaerobic allantoin degradation pathway of *Escherichia coli* includes a global orphan enzyme, oxamic transcarbamylase (OXTCase), which converts oxalurate to carbamoyl phosphate and oxamate. This study found that the *allFGH* (*fdrA*, *ylbE*, and *ylbF*) genes encode OXTCase. The OXTCase activity and kinetics were successfully determined with purified recombinant AllF-AllG-AllH (FdrA-YlbE-YlbF). This OXTCase is a novel transcarbamylase that shows no similarity with known enzymes of the transcarbamylase family such as aspartate transcarbamylase (ATCase), ornithine transcarbamylase (OTCase), and YgeW transcarbamylase (YTCase). In addition, OXTCase activity requires three genes, whereas ATCase is encoded by two genes, and OTCase and YTCase are encoded by a single gene. The current study discovered OXTCase, the last unknown step in allantoin degradation, and this enzyme is a new member of the transcarbamylase group that was previously unknown.

## INTRODUCTION

**Fig 1:**
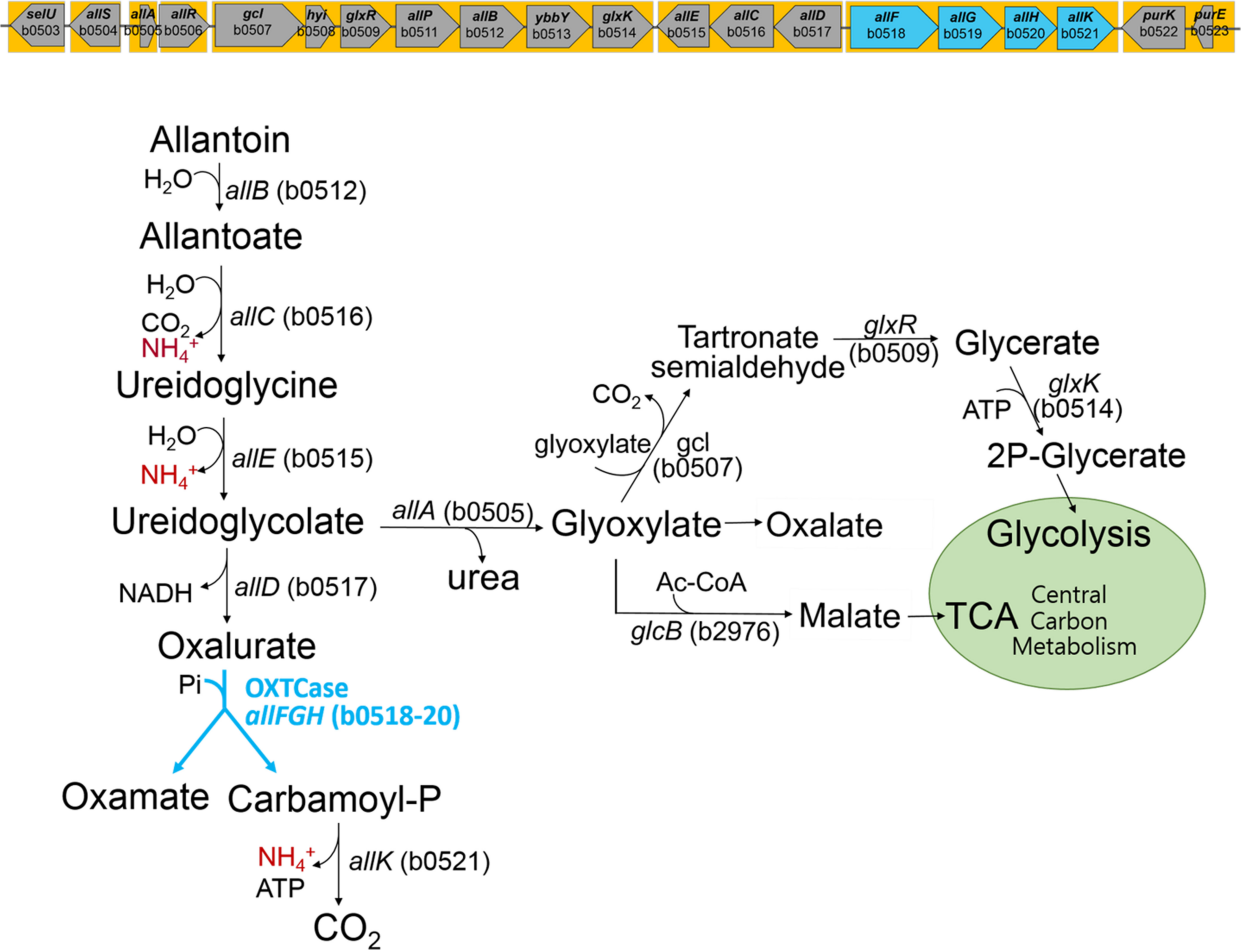
Anaerobic allantoin degradation pathway and the ALL gene cluster for nitrogen assimilation and the carbon anaplerotic process. *allF*/AllF: b0518/Q47208; *allG*/AllG: b0519/P77129; and *allH*/AllH: b0520/P0AAS5.

Allantoin (C_4_H_6_N_4_O_3_) is a nitrogen-rich intermediate in purine degradation (1). *Escherichia coli* and *Streptococcus allantoicus* (*Carnococcus allantoicus*) can degrade and recycle allantoin but only anaerobically (2, 3). *Escherichia coli* uses allantoin as the sole nitrogen source for anaerobic growth (4, 5). Allantoin undergoes hydrolytic ring cleavage (AllB), further hydrolysis (AllC), and oxidation (AllD) processes to release 2 moles of NH_3_ and then becomes ureidoglycolate (C_3_H_6_N_2_O_4_) (6–12) (Fig. 1). Ureidoglycolate is a branch intermediate that produces either glyoxylate or oxalurate (C_3_H_3_N_2_O_4_) (12) (Fig. 1). The former results in a carbon anaplerotic process that allows glyoxylate (C2) to supplement central carbon metabolism such as the TCA cycle or glycolysis (5, 13–16). The conversion of ureidoglycolate to glyoxylate (AllA) releases 1 mole of urea (N2), a waste product of urease-negative *E. coli* (10, 17). The latter results in a nitrogen assimilation (recycling) process; after oxidation of ureidoglycolate to oxalurate, it is converted to carbamoyl phosphate (CP) with the dead-end product oxamate by an oxamic transcarbamylase (OXTCase) (12, 18, 19). CP is a biochemically important molecule that assimilates ammonia into nucleotides and amino acids. Since two ATP molecules are required to form CP from ammonia, reusing CP is energetically advantageous when cells need to synthesize nitrogen-containing molecules. Alternatively, CP is used to produce ATP by carbamate kinase (AllK) under the release of 1 mole of NH_3_ (20). In this pathway, the conversion of oxamate plus CP to form oxalurate is catalyzed by OXTCase; its activity was measured and reported originally in a study with *S. allantoicus* (21). The activity of OXTCase has been shown in crude extracts of *S. allantoicus*, *Streptococcus* D10, and *Streptococcus faecalis* ATCC 11700 (21–23). However, the genes encoding OXTCase have not yet been identified; thus, OXTCase is still classified as a global orphan enzyme (24). *Escherichia coli* is another organism that uses the OXTCase reaction in anaerobic allantoin degradation. While there have been attempts to determine whether the YgeW protein of *E. coli* is an OXTCase, no OXTCase activity has been detected (25).

Based on measurements, OXTCase converts oxalurate to oxamate by phosphorolytic reaction and easily catalyzes reversible reactions (reaction 1) (21, 22, 26, 27). CP can lead to the formation of ATP by catabolic carbamate kinase (CK) (reaction 2), but oxamate is no longer metabolized (18, 20).


(1) $$Oxalurate+{{\text{PO}}_{\text{4}}}^{-}↔Oxamate+CP(OXTCase,EC\;2.1.3.5)$$

(2) CP+ADP→ATP+Carbamate (catabolic CK,EC 2.7.2.2)

Smith et al. (24) used a genome and metabolic context method called candidate genes for orphan enzymes to find the missing enzyme. They predicted that *ylbF* (b0520) was the first candidate gene for OXTCase with an unknown function and that *fdrA* (b0518) was the second candidate as oxamate:CoA ligase, but there was no experimental evidence other than genomic contextual prediction (24). Recently, we reported *fdrA* as a strong candidate gene for OXTCase (28). We found that Δ*fdrA* failed to convert oxalurate to CP and oxamate during anaerobic allantoin degradation, and this degradation was bypassed using an alternative pathway from ureidoglycolate to glyoxylate involving ureidoglycolate lyase (28). Furthermore, the blockage of oxamate formation in Δ*fdrA* was restored by a complement plasmid containing *fdrA,* and a transcriptional fusion *fdrA-lacZ* was induced by allantoin under anaerobic conditions (28). Thus, we expected that *fdrA* encodes the enzyme OXTCase responsible for the step splitting oxalurate into oxamate and CP. However, the activity of OXTCase was not detected when only FdrA was present. In addition, the *fdrA* gene forms one operon with the downstream genes *ylbE*, *ylbF*, and *ybcF* (20) (Fig. 1). In the present study, we show that the OXTCase of *E. coli* is encoded by three genes, *allF* (*fdrA*), *allG* (*ylbE*), and *allH* (*ylbF*), and the OXTCase kinetics were analyzed using purified AllF, AllG, and AllH (FdrA, YlbE, and YlbF). This study is the first to experimentally demonstrate that AllF-AllG-AllH of *E. coli* has OXTCase activity, suggesting that *fdrA*, *ylbE*, and *ylbF* can be renamed *allF*, *allG*, and *allH*, respectively.

## RESULTS

### Allantoin degradation into oxamate is dependent on the three genes *allF* (*fdrA*), *allG* (*ylbE*), and *allH* (*ylbF*)

To determine whether the degradation of allantoin into oxamate is dependent on the three genes *allF, allG,* and *allH*, we investigated anaerobic growth on allantoin (20 mM) as a nitrogen source for 48 h (Table 1). In the wild-type strain, most of the allantoin (19.5 mM) was degraded into oxamate (17.7 mM), and a small fraction was degraded into oxalate (2.4 mM). In all three single-mutant strains, Δ*allF* (LMB076), Δ*allG* (LMB109), and Δ*allH* (LMB110), the conversion to oxamate was interrupted; however, levels of oxalate significantly increased. A previous study showed that ureidoglycolate was bypassed by a glyoxylate shunt due to the blockage of the oxamate-producing step (28). In these single mutants, the interruption of oxamate production tended to be partially complemented by introducing the respective gene on plasmids (^C^*allF*, ^C^*allG*, and ^C^*allH*) (Table 1). However, the effects of gene deletion and complementation on metabolism differed depending on the gene, and there was no apparent consistency. Therefore, we redesigned the experiment to investigate the role of each gene using a triple mutant Δ*allF-allG-allH* (LMB135) strain complemented individually with each single gene on plasmids (^C^*allF*, ^C^*allG*, and ^C^*allH*). The results showed that the oxamate-producing path was not recovered with the single genes (Table 2), which is consistent with the above results (Table 1), in which oxamate production was blocked in the absence of one of the three genes.

**TABLE 1:** Comparison of allantoin consumption and production of oxamate and oxalate by wild-type *E. coli*, strains with a single gene deleted, and strains complemented with a single gene to the strain^*b*^^,^^*c*^^,^^*d*^

		WT	Δ*allF*		Δ*allG*		Δ*allH*	
			None	^C^ *allF*	None	^C^ *allG*	None	^C^ *allH*
Consumption (mM)	Allantoin	19.5 ± 1.0^*a*^	20.0 ± 0.2	20.0 ± 0.3	14.0 ± 0.3	13.9 ± 0.1	19.5 ± 0.4	19.6 ± 0.9
Production (mM)	Oxamate	17.7 ± 1.3	0	4.1 ± 1.5	0	0	1.2 ± 0.3	12.6 ± 1.3
	Oxalate	2.4 ± 1.4	12.0 ± 1.2	8.7 ± 1.1	10.6 ± 0.5	9.2 ± 0.5	10.4 ± 0.2	2.6 ± 0.7

^*a*^ Values represent average ± SD for three replicates.

^*b*^ ^C^Xgene, complementation using pNTR-SD::Xgene.

^*c*^ WT, wild-type; Δgene, gene deletion mutant.

^*d*^ They were anaerobically cultured in glycerol (50 mM), dimethyl sulfoxide (50 mM), and allantoin (20 mM) in nitrogen-deficient M9 media for 48 hours. The strain with genes deleted from the genome was complemented by a plasmid with the corresponding genes.

**TABLE 2:** Comparison of allantoin consumption and production of oxamate and oxalate by a strain with *allFGH* deleted and strains complemented with a single gene to the strain^*b*^^,^^*c*^^,^^*d*^

Strain		Δ*allFGH*			
Plasmid		None	^C^ *allF*	^C^ *allG*	^C^ *allH*
Consumption (mM)	Allantoin	13.3 ± 2.7^*a*^	11.4 ± 0.8	11.7 ± 1.2	11.8 ± 0.1
Production (mM)	Oxamate	0	0	0	0
	Oxalate	8.0 ± 2.5	6.6 ± 0.6	7.0 ± 0.4	5.5 ± 0.1

^*a*^ Values represent average ± SD for three replicates.

^*b*^ ^C^Xgene, complementation using pNTR-SD::Xgene.

^*c*^ Δgene, gene deletion mutant.

^*d*^ They were anaerobically cultured in glycerol (50 mM), dimethyl sulfoxide (50 mM), and allantoin (20 mM) in nitrogen-deficient M9 media for 48 hours. The strain with genes deleted from the genome was complemented by a plasmid with the corresponding genes.

The complementation of the triple mutant by multiple gene combinations on plasmids [^C^(*allFGH*), ^C^(*allFG*), and ^C^(*allGH*)] was investigated during anaerobic growth on allantoin (40 mM) as the nitrogen source for 72 h. Complementation with all three genes (*allFGH*) restored the oxamate level of the triple mutant to that of the wild-type strain (Table 3). Complementation with *allFG* or *allGH* showed a lower level of restoration, at 7% and 16% of the wild-type oxamate level, respectively (Table 3).

**TABLE 3:** Comparison of allantoin consumption and production of oxamate and oxalate by wild-type *E. coli* and *ΔallFGH* strains according to plasmid complementation containing genes deleted from the genome during anaerobic growth in glycerol (50 mM), dimethyl sulfoxide (50 mM), and allantoin (40 mM) in nitrogen-deficient M9 media for 72 h^*b*^^,^^*c*^^,^^*d*^

Strain		WT	Δ*allFGH*			
Plasmid		None	None	^C^(*allFGH*)	^C^(*allFG*)	^C^(*allGH*)
Consumption (mM)	Allantoin	40.6 ± 0.6^*a*^	25.5 ± 3.4	40.6 ± 0.4	14.3 ± 7.7	40.9 ± 0.6
Production (mM)	Oxamate	38.3 ± 5.6	0	37.5 ± 1.1	2.9 ± 3.3	6.2 ± 3.0
	Oxalate	2.8 ± 0.8	17.9 ± 2.7	0.7 ± 1.2	5.3 ± 4.3	14.8 ± 1.2

^*a*^ Values represent average ± SD for three replicates.

^*b*^ ^C^Xgene, complementation using pNTR-SD::Xgene.

^*c*^ WT, wild-type; Δgene, gene deletion mutant.

^*d*^ The strain with genes deleted from the genome was complemented by a plasmid with the corresponding genes.

Taken together, these findings indicate that the oxamate-producing step catalyzed by OXTCase involves an enzyme encoded by the three genes *allF, allG*, and *allH*.

### Detection of oxalurate

To demonstrate that AllF-AllG-AllH (FdrA-YlbE-YlbF) forms the OXTCase enzyme responsible for converting oxalurate to oxamate and carbamoyl phosphate, detecting the substrate oxalurate is essential. However, oxalurate could not be used as a standard substance due to its unavailability, and the enzyme reaction was performed in the reverse direction: oxamate + CP → oxalurate. Previous studies also investigated OXTCase activity using this reverse reaction (21). We measured OXTCase activity based on the stoichiometry of the reaction, enabling the calculation of an apparent equilibrium constant using the equilibrium concentration of oxalurate and the initial concentrations of oxamate and CP (23). The calculations were based on values of oxalurate at equilibrium, so the consumption of oxamate used as a substrate was considered equal to the amount of oxalurate produced by the reaction (21). A study using *S. allantoicus* also showed that oxamate formation correlated well with oxalurate decomposition (26).

**Fig 2:**
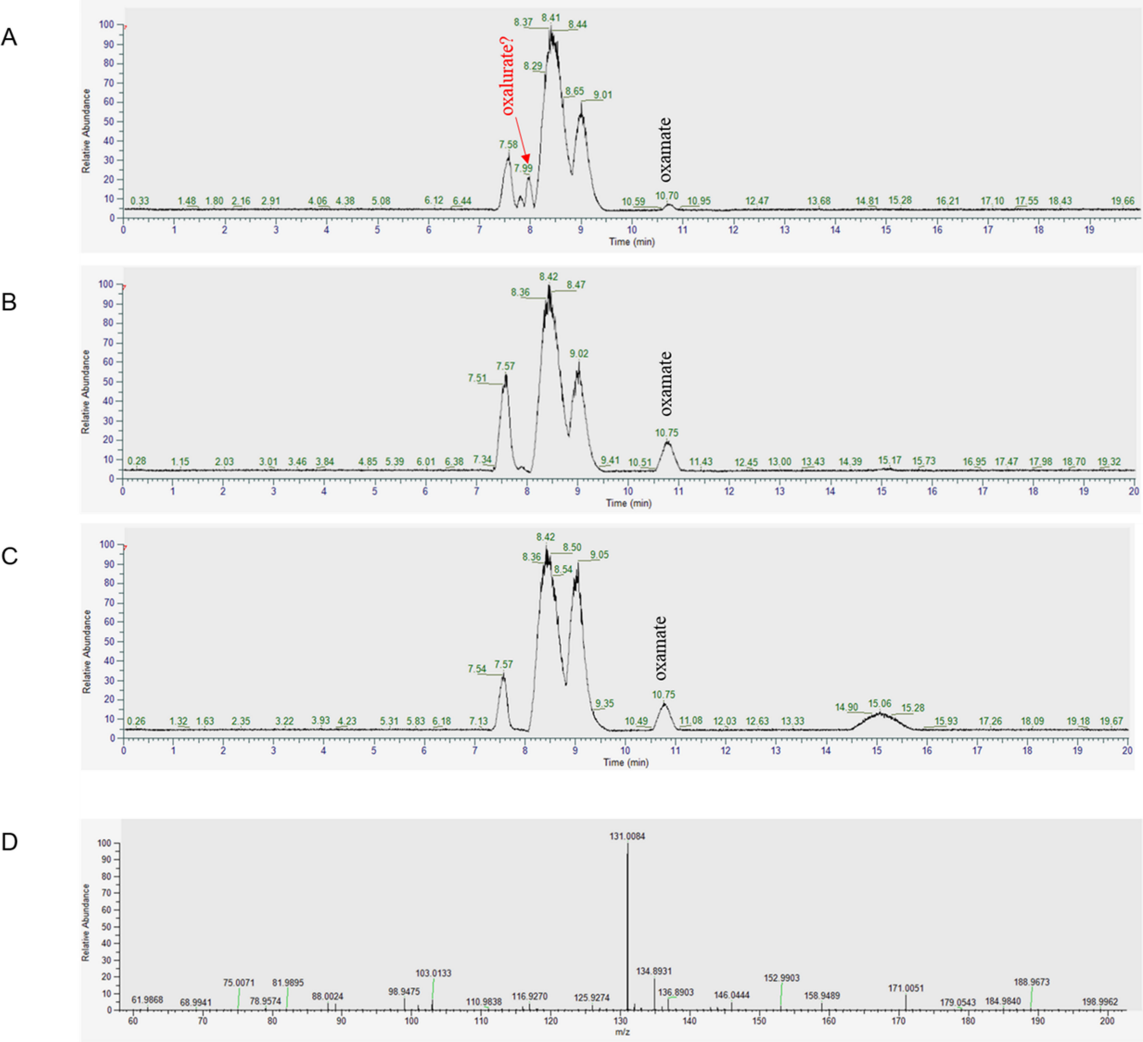
Detection of oxalurate produced by oxamic transcarbamylase of *E. coli*. Total ion chromatogram of a reaction sample using cell lysate of (**A**) the wild-type strain, (**B**) the Δ*allF* strain, and (**C**) a buffer control. (**D**) Mass of the new peak that occurred at 7.99 min in the analysis in panel **A**. The wild-type and Δ*allF* strains were grown anaerobically on allantoin, and the crude lysate was used as an enzyme. The reaction mixture contained 5 mM of oxamate, 5 mM of carbamoyl phosphate, 10 mM of MgCl_2_, 100 mM of Tris buffer (pH 9.0), 0.1 mL lysates, and distilled water to a volume of 1.0 mL.

Crude lysates of wild-type and Δ*allF E. coli* cells grown anaerobically on allantoin were used for the OXTCase reaction and analyzed by HPLC. The lysate of wild-type cells converts oxamate into oxalurate, so the newly generated substance at 7.6 min, compared with Δ*allF* or the buffer control, is likely to be oxalurate (Fig. S1A). We analyzed the same reaction using a total ion chromatogram (TIC), which allows comprehensive detection. The new peak (retention time 7.99 min) appeared in the reaction of the wild-type strain (Fig. 2A) compared with that of the Δ*allF* strain or a buffer control (Fig. 2B and C). The mass of the new peak was analyzed by LC-MS as 131.0084, which corresponds to the molecular weight of oxalurate (Fig. 2D).

The OXTCase assay was performed using the purified recombinant AllFGH, and HPLC analysis showed the conversion of oxamate (at 10.1 min) to a new peak (7.6 min) (Fig. S1B), which was similar to that of the crude lysate (Fig. S1A). The new peak formed by purified AllFGH was evaluated using ^13^C NMR and ^15^N HSQC spectra (Fig. S2). The ^13^C NMR spectrum shows the carbonyl group region (150–175 ppm) typical of esters, acids, and amides; the carbonyl group (*) newly formed by carbamoyl transfer appeared at 157.1 ppm (Fig. S2A). The two singlet peaks at 169.1 and 167.5 ppm are derived from oxamate (Fig. S2A through C), and the large singlet peak at 162.5 ppm is derived from CP (Fig. S2A, B, and D). The ^1^H-^15^N HSQC NMR spectrum also revealed the presence of two newly formed H-N bonds (*) of oxalurate at 5.68/76.89 and 7.10/107.17 ppm, which had been shifted from those of oxamate and CP (Fig. S2E and F). The peaks newly generated in the ^13^C and ^1^H-^15^N HSQC NMR spectra are presumed to originate from oxalurate with two N-H bonds and carbonyl groups. However, due to the limited quantity of the product generated from the reverse enzymatic reaction and the measured samples being mixtures with substrates and product, there were some limitations in obtaining clear NMR spectra to assign all the peaks of the molecular structure; these results are provided in Fig. S2.

In summary, the aim of these experiments was to validate that AllFGH forms the OXTCase enzyme by detecting oxalurate using a reverse enzyme reaction due to the unavailability of oxalurate. HPLC, TIC, LC-MS, and NMR analyses using both cell lysates and purified recombinant AllFGH showed that oxamate and CP were converted to oxalurate. As the reverse reaction was employed, the quantity of generated oxalurate was relatively limited, resulting in constraints on clear spectra. Nevertheless, the analysis data confirmed the activity of AllFGH as an OXTCase.

### Purification of 6×His-AllF-AllG-AllH-6×His

**Fig 3:**
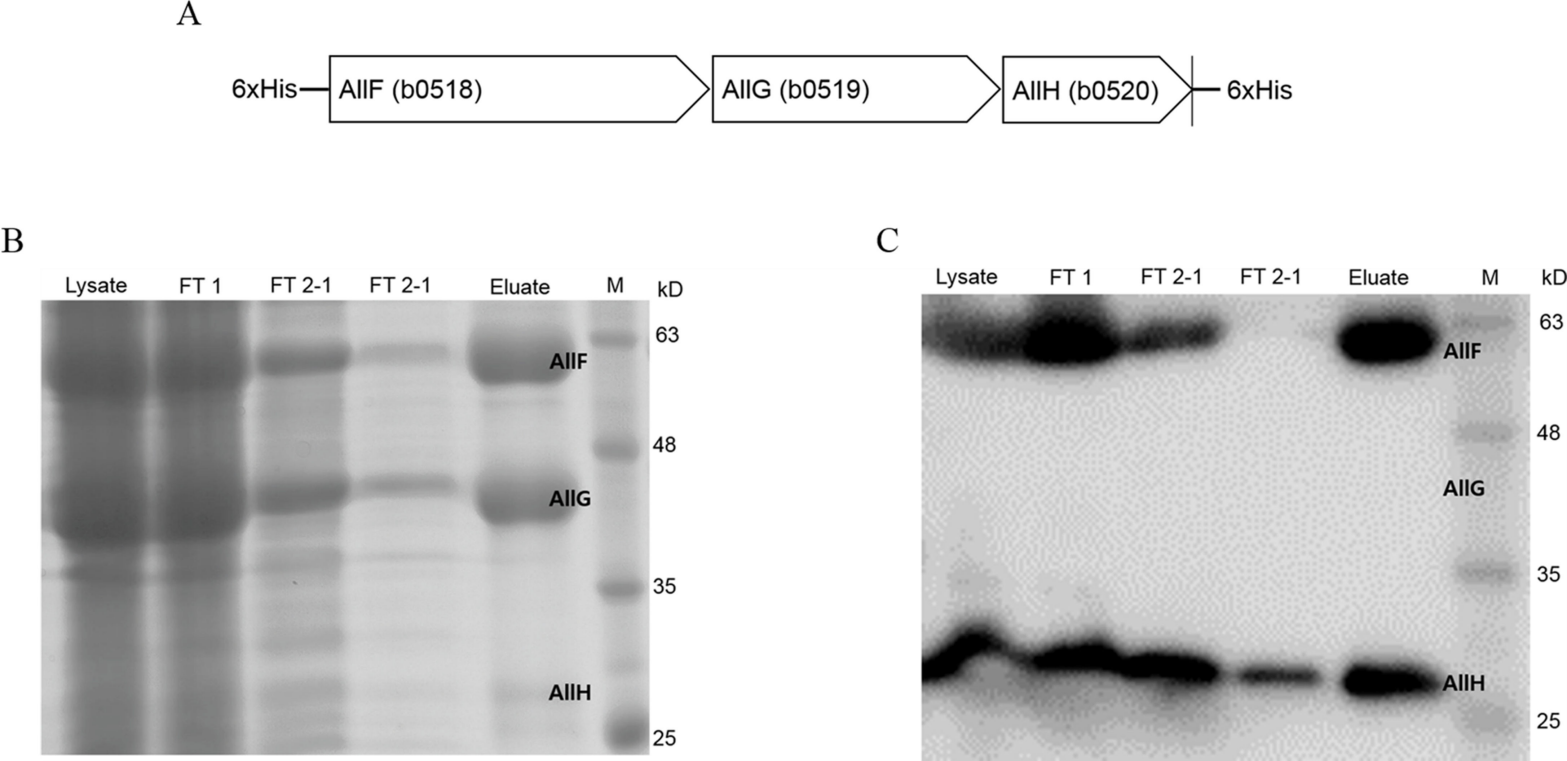
Purification of AllF-AllG-AllH. The 6×His-AllF, AllG, and AllH-6×His proteins were overproduced in BL21(DE3) cells expressing pMB191 [pET30b::fdrAylbEylbF (*allFGH*)] and purified. (**A**) Schematic diagram of AllF-AllG-AllH with 6× histidines at the N- and C-termini, (**B**) SDS-PAGE, and (**C**) western blot. Sizes of polypeptides: 6×His-AllF, 62 kDa; AllG, 46 kDa; and AllH-6×His, 31 kDa. Lysate, crude lysate; FT 1, flow-through fraction; FT 2-1 and FT 2-2, wash fraction; eluate: elution fraction of AllFGH; and M, protein ladder.

For overexpression and purification of the protein composed of AllF-AllG-AllH, the three genes *allFGH* were cloned into pET30b with a 6×His-tag at both the N- and C-termini (pMB191) (Table S1). The successful overproduction of 6×His-AllF (62 kDa), AllG (46 kDa), and AllH-6×His (31 kDa) was confirmed by SDS-PAGE (Fig. 3A and B), and the AllF and AllH bands with His-tags were confirmed on western blot (Fig. 3C). In contrast, when we cloned the three genes using a single 6×His-tag at the C-terminus (pMB190) (Table S1), only a small fraction of the His-tagged AllH was detected, indicating that AllF and AllG were washed out (data not shown). Therefore, we proceeded with enzyme activity assays using purified proteins obtained from cloning with His tags attached to both the N- and C-termini (pMB191).

### Optimizing the *E. coli* OXTCase assay: pH, temperature, and Mg^2+^ ion influence

**Fig 4:**
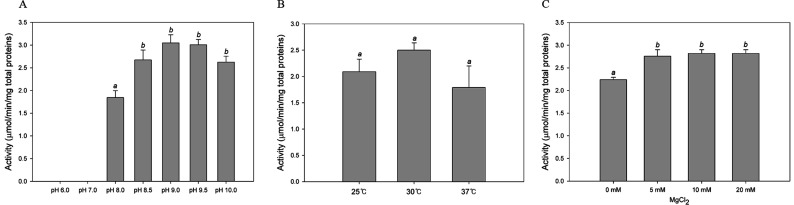
Oxamic transcarbamylase activity of crude lysates of AllFGH-overproducing-*E. coli* depending on pH (**A**), temperature (**B**), and magnesium ions (**C**). Reaction mixture (**A**) contained 20 mM of oxamate, 20 mM of carbamoyl phosphate, 10 mM of MgCl_2_, 100 mM of Tris buffer (pH 6.0–10.0), 0.1 mL lysates, and distilled water to a volume of 1.0 mL; reaction mixture (**B**) contained 20 mM of oxamate, 20 mM of CP, 10 mM of MgCl_2_, 100 mM of Tris buffer (pH 9.0), 0.1 mL lysates, and distilled water to a volume of 1.0 mL. The reaction proceeded at 25°C, 30°C, and 37°C, respectively; the reaction mixture (**C**) contained 20 mM of oxamate, 20 mM of CP, MgCl_2_ (0–20 mM), 100 mM of Tris buffer (pH 9.0), 0.1 mL lysates, and distilled water to a volume of 1.0 mL. The reaction shown in panels **A** and **C** was incubated at 30°C. Identical letters in respective conditions indicate no significant differences at *P* < 0.05 as calculated in SPSS PAWS Statistics with one-way analysis of variance and Duncan’s multiple comparisons. Values were determined from three replicates. Error bars indicate standard deviations.

The optimal conditions for the OXTCase activity of *E. coli*, i.e., pH, temperature, and Mg^2+^ ion concentration, were determined using crude lysates of cells overproducing AllF-AllG-AllH. The activity for oxalurate production was expressed as the amount of oxamate consumption per minute forper milligram of total proteins. First, the OXTCase activity was measured in the pH range of 6.0–10.0. No enzyme activity was detected at pH 6.0 and 7.0 (Fig. 4A). Activity increased in the pH range of 8.0–9.0, peaking at pH 9.0, indicating the optimal pH value for the *E. coli* OXTCase activity (Fig. 4A). This is similar to the optimal activity observed for the *S. allantoicus* OXTCase at pH 8.5 (21). The optimal temperature for *E. coli* OXTCase activity was determined to be 30°C, tested over a range from 25°C to 37°C (Fig. 4B). Valentine and Wolfe (27) reported the dependency of OXTCase activity on Mg^2+^ ions. Testing Mg^2+^ ion concentrations at the optimal pH (pH 9.0) and temperature (30°C) revealed saturation of *E. coli* OXTCase activity at 10 mM Mg^2+^ ions (Fig. 4C). In contrast, the *S. allantoicus* OXTCase functions optimally at 2.5 mM Mg^2+^ ions (21).

**Fig 5:**
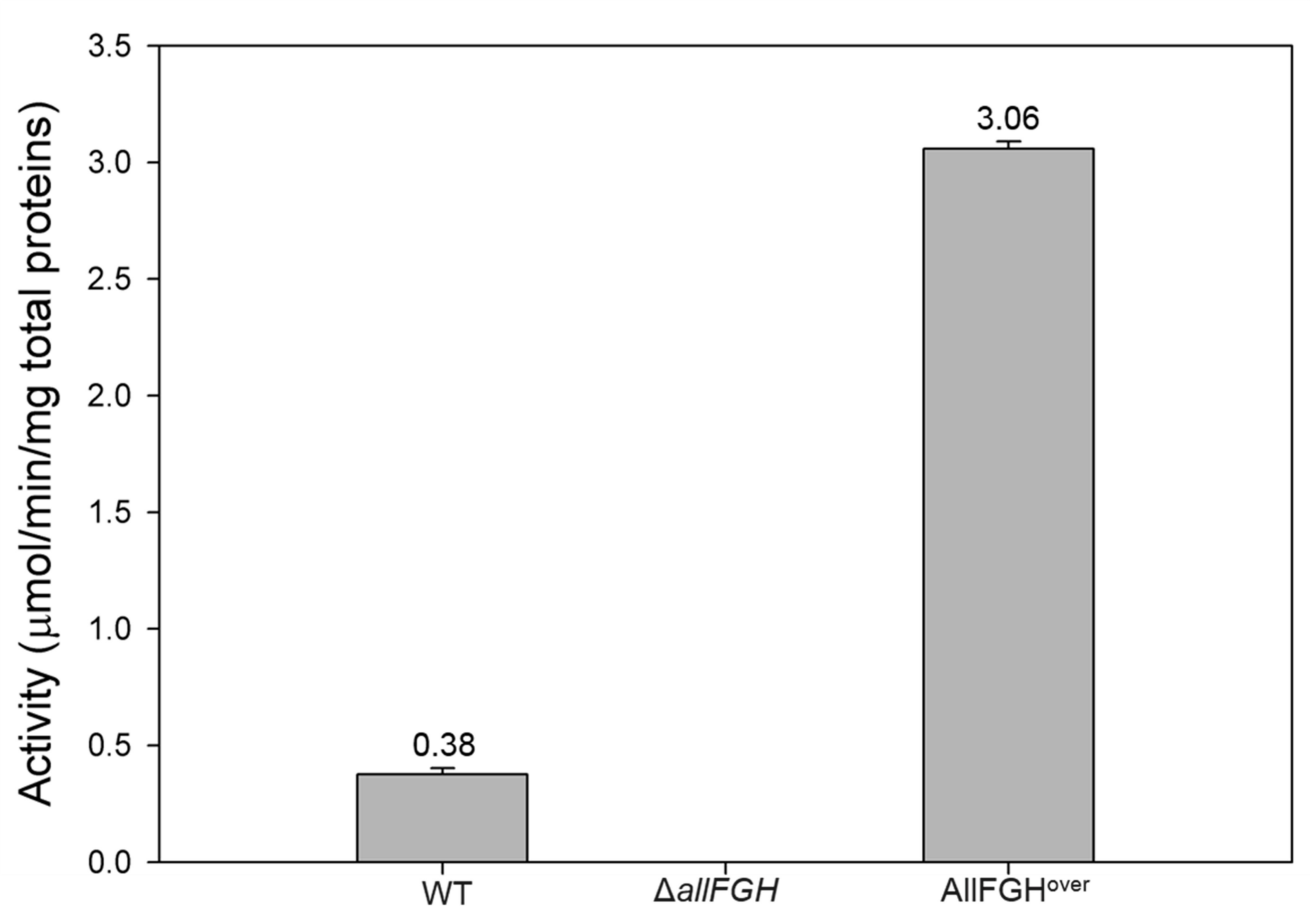
Evaluation of the overproduced AllF-AllG-AllH of *E. coli* for oxamic transcarbamylase activity. Wild-type and triple-mutant (Δ*allFGH*) *E. coli* were anaerobically grown on allantoin as a sole nitrogen source in nitrogen-deficient M9. The AllFGH-overproducing (AllFGH^over^) *E. coli* was aerobically cultivated under induction conditions. The enzyme reaction was started by the addition of cell lysates (0.1 mL) to the reaction mixture, which contained 20 mM of oxamate, 20 mM of carbamoyl phosphate, 10 mM of MgCl_2_, 100 mM of Tris buffer (pH 9.0), and distilled water to a volume of 1.0 mL. Values were determined from three replicates. Error bars indicate standard deviations.

Under the optimized conditions, OXTCase activity using the cell lysate of the overproduced AllF-AllG-AllH (AllFGH^over^) was 3.06 µmol/min/mg total protein. This was eight times higher than the activity of the cell lysate of the wild-type strain, which was 0.38 µmol/min/mg total protein (Fig. 5). This wild-type level was similar to the 0.6 µmol/min/mg protein of the OXTCase activity in crude extracts of *S. allantoicus* (21). These data indicate that the recombinant protein 6 × His-AllF-AllG-AllH-6×His functions as an enzyme.

### Characterization of OXTCase activity using the purified AllF-AllG-AllH protein

**Fig 6:**
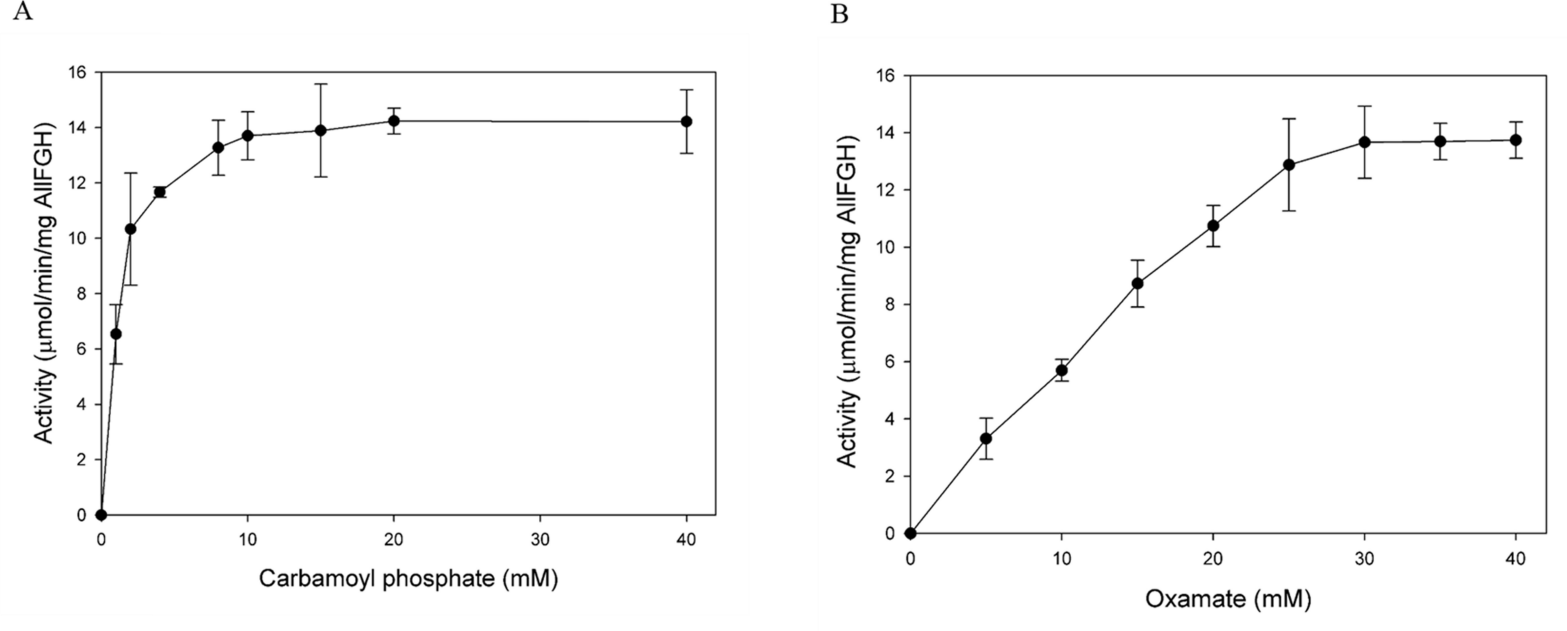
Oxamic transcarbamylase activity of purified AllFGH depending on the concentrations of carbamoyl phosphate (**A**) and oxamate (**B**). Each reaction mixture contained oxamate, CP, 10 mM of MgCl_2_, 100 mM of Tris buffer (pH 9.0), 100 nM AllFGH, and distilled water to a volume of 1.0 mL; keeping oxamate fixed at 40 mM, the activity was measured as the concentration of CP was changed from 0 to 40 mM. The kinetic parameters *V*_max_ (maximum velocity) and *K*_*M*_ (Michaelis-Menten constant) for oxamic transcarbamylase were calculated from the Lineweaver-Burk equation and Michaelis-Menten equation, respectively. Values were determined from three replicates. Error bars indicate standard deviations.

The above recombinant protein AllFGH was purified and used for an enzyme assay at a concentration of 100 nM. Here, the molecular mass of OXTCase was assumed to be 139 kDa, which was calculated considering it as a heterotrimer composed of three monomers [(AllF)_1_ (AllG)_1_ (AllH)_1_]. The size of the monomers 6× His-AllF, AllG, and AllH-6×His were 62, 46, and 31 kDa, respectively. The OXTCase kinetics using AllFGH was investigated under the optimized conditions in a buffer of pH 9.0 with 10 mM Mg^2+^ at 30°C for 10 min. Since the OXTCase analysis was conducted in the reverse direction, the substrates were oxamate and CP (EC 2.1.3.5), so the OXTCase activity was measured depending on the concentration of each of the two substrates. The activity was expressed as the amount of oxamate consumption per minute per milligram of AllFGH protein. When analyzing the CP concentration as a variable, the oxamate concentration was fixed at 40 mM, a concentration that would not affect the activity. For CP, the *V*_max_ (*V*_max_CP_) was 15.4 µmol/min/mg AllFGH, and the *K*_*M*_ value (*K*_*M*_CP_) was 1.3 mM (Fig. 6A). From this, the *k*_cat_ for CP was calculated as 35.5 s^−1^ and the *k*_cat_*/K*_*M*_ as 27.3 s^−1^·mM^−1^ (Table 4). For oxamate, the *V*_max_ (*V*_max_OX_) was 27.0 µmol/min/mg AllFGH and the *K*_*M*_ value (*K*_*M*_OX_) was 36.9 mM (Fig. 6B). Then, *k*_cat_ for oxamate was calculated as 58.5 s^−1^ and *k*_cat_*/K*_*M*_ as 1.6 s^−1^·mM^−1^ (Table 4). Despite the similar levels of *V*_max_ for the two substrates, the *K*_*M*_ values were significantly different, and the affinity for CP was about 30 times higher than that for oxamate. Accordingly, the *k*_cat_ values for the two substrates are similar, but the *k*_cat_ */K*_*M*_ value for CP was approximately 20 times higher than that for oxamate.

**TABLE 4:** Kinetic parameters of oxamic transcarbamylase using purified AllFGH for carbamoyl phosphate and oxamate

	Carbamoyl phosphate	Oxamate
*K*_*M*_ (mM)	1.3	36.9
*V*_max_ (μmol/min·mg OXTCase)	15.4	27.0
*k*_cat_ (s^−1^)	35.5	58.5
*k*_cat_ */K*_*M*_ (s^−1^·mM^−1^)	27.3	1.6

## DISCUSSION

Enzymes of the transcarbamylase family catalyze the transfer of a carbamoyl group from CP to an amino group of the second substrate. The best-studied members of this family are aspartate transcarbamylase (ATCase) and ornithine transcarbamylase (OTCase), which are present in most organisms (29) (Table 5). ATCase catalyzes the first step of *de novo* biosynthesis of pyrimidine by transfer of a carbamoyl group from CP to aspartate to yield carbamoyl aspartate (30). OTCase catalyzes anabolic or catabolic reactions. Anabolic OTCase catalyzes the carbamylation of ornithine to citrulline in the urea cycle or arginine biosynthesis, whereas catabolic OTCase is used in ATP generation in the arginine deiminase pathway, which is found only in bacteria (29, 31). In addition, newly discovered transcarbamylases have recently been reported in some bacteria, such as YgeW in *E. coli* (25). Li et al. discovered the gene *ygeW*, which was predicted to encode a novel transcarbamylase, and analyzed its crystal structure. The results demonstrated that YgeW has a structural singularity that distinguishes it from the known transcarbamylases (25). Li et al. predicted YgeW to be an OXTCase, but no transcarbamylase activity was detected in tests with more than 20 oxamate- or allantoin-related compounds as potential substrates; therefore, YgeW is a transcarbamylase with unknown function (UTCase) (25). In the present study, we found a new type of transcarbamylase, the OXTCase, in an inductive manner. We noted that single nucleotide polymorphisms in the *fdrA* (now renamed as *allF*) gene changed the metabolic flow into the glyoxylate shunt (28). However, in scientific databases, *fdrA* had been annotated as a gene encoding a putative acyl-CoA synthetase, oxamate:CoA ligase (Pfam 00549), or multicopy suppressor of dominant negative ftsH mutations (32), and *ylbE* and *ylbF* were listed as having unknown functions. This indicates the lack of similarity in the protein sequence to enzymes in the transcarbamylase family, which could be the reason why FdrA-YlbE-YlbF (AllF-AllG-AllH) has not been predicted as an OXTCase.

**TABLE 5:** Enzymes of the transcarbamylase family in *E. coli^a^*

	ATCase		OTCase		YgeW	OXTCase		
			OTCase-1	OTCase-2				
Gene	*pyrB-pyrI* (b4245-4244)		*argI* (b4254)	*argF* (b0273)	*ygeW* (b2870)	*allF-allG-allH* (b0518-0520)		
Length	936 bp	462 bp	1,005 bp	1,005 bp	1,191 bp	1,668 bp	1,260 bp	816 bp
	311 aa	153 aa	334 aa	334 aa	396 aa	555 aa	419 aa	271 aa
Subunit composition	[(PyrB)_3_]_2_[(PyrI)_2_]_3_		[ArgI]_3_	[ArgF]_3_	[YgeW]_3_	[(AllF)_2_]_*x*_[(AllG)_2_]_*x*_[(AllG)]_*y*_ , where *x* > *y*		
Reaction	L-Asp + CP → N-carbamoyl-L-Asp + P_i_ + H^+^		L-Orn + CP ↔ L-Cit + P_i_ + H^+^		Unknown	Oxamate + CP ↔ Oxalurate + P_i_		
References	(33–38)		(39–41)		(25)	This study		

^*a*^ L-Asp, L-aspartate; P_i_, inorganic phosphate; L-Orn, L-ornithine; and L-Cit, L-citrulline.

Another interesting point is that the activity of OXTCase requires three genes, and no other transcarbamylase is encoded by three genes (Table 5). The ATCase of *E. coli* is encoded by *pyrI-pyrB* and composed of two catalytic trimers [(PyrB)_3_]_2_ and three regulatory dimers [(PyrI)_2_]_3_. The other transcarbamylases of *E. coli,* OTCase-1, OTCase-2, and YgeW, are encoded by single genes and form a trimer. In addition, the genes *argI* (OTCase-1), *argF* (OTCase-2), *ygeW* (YgeW), and *pyrB* (the catalytic subunit of ATCase) form the paralog gene group No. 84 (total four members) in the EcoCyc *E. coli* Database (https://ecocyc.org/). AlphaFold2 provided by ColabFold v1.5.3 (https://colab.research.google.com/github/sokrypton/ColabFold/blob/main/AlphaFold2.ipynb?authuser=1#scrollTo=kOblAo-xetgx) was used to predict whether each of the three subunits of the OXTCase, AllF, AllG, and AllH, forms a multimer. The Predicted Aligned Error (PAE) plots indicate that AllF and AllG would form homodimers, (AllF)_2_ and (AllG)_2_, respectively, and that AllH would be a monomer, (AllH)_1_, which can be noted by a consistent pattern within the plots (Fig. S3), where PAE is the indicator of the reliability of prediction for multimer formation. Among these subunits, AllF has been determined to be a homodimer through crystallization structure analysis (PDB ID 3DMY; https://doi.org/10.2210/pdb3DMY/pdb). No further quaternary structures were provided by AlphaFold-multimer; thus, it is difficult to predict how many dimers or monomers (AllF)_2_, (AllG)_2_, or (AllH)_1_ form the OXTCase. Moreover, since AllF and AllG appeared in similar amounts on SDS-PAGE (Fig. 3B), we predict that the numbers of (AllF)_2_ and (AllG)_2_ will be the same. However, such results were not observed on western blot (Fig. 3C). Accordingly, it can be estimated that OXTCase consists of [(AllF)_2_]_*x*_[(AllG)_2_]_*x*_[(AllG)]_*y*_, where *x* > *y* (Table 5). Therefore, this OXTCase is a new enzyme that differs in the number of composition subunits. Subsequent studies on its protein structure will soon be conducted.

The binding of oxalurate, oxamate, and carbamoyl phosphate to the OXTCase subunits (AllF)_2_, (AllG)_2_, and AllH was simulated using the structure-based docking database CB-DOCK2 server (https://cadd.labshare.cn/cb-dock2/php/index.php) (Fig. S4). The previously characterized enzymes AllB (allantoin amidohydroase), AllC (allantoate amidohydrolase), and AllE (ureidoglycine aminohydroase) and their substrates allantoin, allantoate, and ureidoglycolate were used as a positive reference for the protein-ligand docking score, which is called a Vina score (Table S2). AllB (tetramer), AllC (dimer), AllE (dimer), and AllF (dimer) adopted crystallized structures from RCSB PDB, whereas AllG (dimer) and AllH (monomer) obtained putative structures from AlphaFold-multimer (Fig. S4). The result of CB-DOCK2 ranked the best binding mode in each pocket and the potential binding sites of the query ligand according to the Vina score (kcal/mol) that automatically calculates the grid maps and clusters the results (42). The Vina scores of the positive reference (AllB)_4_-allantoin, (AllB)_4_-allantoate, (AllC)_2_-allantoate, and (AllE)_2_-S-ureidoglycolate ranged from −4.5 to −6.4 (kcal/mol) (Table S2). The docking scores for oxalurate to (AllF)_2_, (AllG)_2_, and AllH were -6.4,–6.4, and −5.6 (kcal/mol), respectively, indicating highly reliable values for ligand binding (Table S2; Fig. S4). In contrast, the docking scores for oxamate or CP to each protein decreased to −4.3 to −5.2 (kcal/mol), with particularly low energies observed for oxamate (Table S2), which is consistent with the high *K*_*M*_ value (36.9 mM) of AllFGH for oxamate (Fig. 6B). The positions (centers) represent where oxalurate, CP, or oxamate dock to each protein match: (AllF)_2_-oxalurate, -CP, or -oxamate; (AllG)_2_-oxalurate, -CP, or -oxamate; AllH-oxalurate, -CP, or -oxamate (Table S2; Fig. S3). Therefore, when they form a complex, it can be expected that the docking centers of each of these three subunits will overlap.

Urease-negative enteric bacteria such as *E. coli*, *Salmonella enterica*, and *Citrobacter freundii* have homologous genes encoding OXTCase (AllF, AllG, and AllH) and carbamate kinase (AllK), but the urease-positive bacterium *Klebsiella pneumonia* has no genes for these enzymes (Table S3). In urease-negative bacteria, OXTCase (and carbamate kinase) is the only route through which the nitrogen contained in ureidoglycolate or oxalurate can be further assimilated (recycled) (Fig. 1), while urease-positive bacteria can recycle ammonia from urea. The *E. coli* O157:H7 genome contains the genes for OXTCase and carbamate kinase through urease positivity, probably because the genes for urease were later acquired (Table S3).

Taken together, our results demonstrate that the novel protein composed of AllF-AllG-AllH (b0518-20) has OXTCase activity and completes the anaerobic allantoin degradation of *E. coli* together with catabolic carbamate kinase (AllK, b0521) (20).

## MATERIALS AND METHODS

### Strains and allantoin fermentation

The strains and plasmids used in this study are shown in Table S1. Strains were grown on glycerol (50 mM), dimethyl sulfoxide (50 mM), and allantoin (20–40 mM) in nitrogen-deficient M9 minimal medium (_ND_M9) (43) at 37°C under anaerobic conditions with a 95% N_2_ + 5% H_2_ gas mixture. The addition of 0.2 mM isopropyl β-D-1-thiogalactopyranoside (LPS Solution, Republic of Korea) was used for complementary fermentative evaluation of plasmids with specific genes.

### Construction of gene-deleted strains and plasmids

Genes in the genome of *E. coli* K-12 MG1655 (F^–^ λ^–^ *ilvG*^–^ *rfb-50 rph-1*) were deleted using the method of Datsenko and Wanner (44). For insertional inactivation, the PCR product of the *catR* chloramphenicol resistance gene from pKD3 was used and flanked by FRT sequences. Primers contain parts of the regions adjacent to FRT and the 5′ and 3′ regions of the amplified genes (Table S4). The strains in which *allG, allH,* and *allFGH* were replaced by *catR* were designated LMB109, LMB110, and LMB135 (Table S1).

The pNTR-SD::gene plasmid was used to evaluate the supplementary fermentation, and the pET30b plasmid was used to produce AllFGH (Table S1). Genes were amplified from *E. coli* MG1655 genomic DNA using specific primers (Table S4). The amplified fragments were cloned into specific restriction sites of the plasmid. The plasmid pMB190 was designed to have six histidines at the C-terminal end of the AllFGH. A site-directed mutagenesis kit was used to attach six additional histidines to the front of AllF following the manufacturer’s manual (E0552, New England BioLabs Inc., USA). The cloned product was transformed into DH5α cells, confirmed through sequencing (Cosmogenetech Co., Ltd., Republic of Korea), and used in experiments.

### Expression and purification of AllFGH

The AllFGH overexpression plasmid pMB191, which includes 6× His at the N-terminal of AllF, was transformed into BL21(DE3) cells and aerobically grown in LB Broth (Difco, USA) to OD_600_ of approximately 0.4 at 37°C. Induction was performed with 0.2 mM IPTG for 4 h at 30°C. For purification of the histidine-tagged proteins, cell pellets were suspended in lysis buffer [20 mM tris(hydroxymethyl)aminomethane (Tris) buffer pH 8.3, 0.5% triton X-100, and 1 mg/mL lysozyme], and cell disruption was achieved by six repetitions of vortexing with acid-washed glass beads (G1145, Sigma-Aldrich, USA) and freezing for 5 min. Cell debris was removed by centrifugation (13,000 rpm, 4°C, 20 min), and the supernatant was collected. The purification of AllFGH was performed via affinity chromatography with Ni-NTA Spin Columns (31014, Qiagen, Germany). The Ni-NTA spin column was equilibrated with 600 µL NPI-10 buffer (50 mM NaH_2_PO_4_, 300 mM NaCl, and 10 mM imidazole, pH 8.0) and centrifuged for 2 min at 890 × *g*. The cleared lysate was loaded onto the pre-equilibrated Ni-NTA spin column and centrifuged for 5 min at 270 × *g*. The Ni-NTA spin column was twice washed with 600 µL NPI-20 buffer (50 mM NaH_2_PO_4_, 300 mM NaCl, and 20 mM imidazole, pH 8.0) and centrifuged for 2 min at 890 × *g*. The protein was eluted with NPI-500 buffer (50 mM NaH_2_PO_4_, 300 mM NaCl, and 500 mM imidazole, pH 8.0) by centrifuging for 2 min at 890 × *g*, and the eluate was collected. Protein concentrations were determined with the Bradford method using Protein Assay Dye (5000006, Bio-Rad, USA). A standard curve was generated using bovine serum albumin.

### Western blotting

Elution fractions were subjected to SDS-PAGE (Bio-Rad) using a 12% running gel. Electrophoretic transfer to an Amersham Hybond P western blotting membrane (GE Health, USA) was performed with a Trans-Blot SD Semi-Dry Transfer Cell (Bio-Rad). After transfer (15 V, 1 h 20 min), the membrane was blocked for 1 h at room temperature. Then, the membrane was incubated overnight at 4°C with 6× His-tag monoclonal primary antibody (MA1-21315, Invitrogen, USA), diluted 1:2,000. Horseradish peroxidase-coupled goat anti-mouse secondary antibody (G21040, Invitrogen) was diluted 1:100,000 and incubated with the membrane at room temperature for 1 h. Dyne ECL Pico Plus (GBE-P200, Dynebio, Republic of Korea) was used to detect peroxidase activity. Image development was performed using an ImageQuant800 (Amersham, USA).

### Assay of oxamic transcarbamylase

Specific activities of OXTCase were expressed as the amount of enzyme that catalyzes the consumption of 1 µmole of oxamate per minute per milligram of protein. Kinetics was measured using 100 nM AllFGH. The calculation is based on the assumption that OXTCase is composed of one subunit of each AllF, AllG, and AllH. Tris buffer pH (6.0–10.0), Mg^2+^ ion concentration (0–20 mM), and reaction temperature (25°C, 30°C, and 37°C) were evaluated. The standard mixture specified for the OXTCase reaction contained oxamate (5–40 mM) (O3750, Sigma-Aldrich), CP (5–40 mM) (C5625, Sigma-Aldrich), 10 mM of MgCl_2_, 100 mM of Tris buffer (pH 9.0), enzyme, and distilled water to a 1.0 mL volume. Crude lysates were added as 10% of the reaction volume. The reaction was initiated by the addition of the enzyme and was continued for 10 min at 30°C. The reaction was stopped by heating at 95°C for 5 min. The amount of oxamate in the reactant was measured with HPLC.

The Michaelis-Menten constant (*K*_*M*_), maximum velocity (*V*_max_), catalytic constant (*k*_cat_), and catalytic efficiency (*k*_cat_/*K*_*M*_) of oxamate and CP were calculated using the Michaelis-Menten equation and Lineweaver-Burk equation, respectively.

### HPLC analysis

Culture supernatants and OXTCase reactants were analyzed using a Hitachi LaChrom Elite HPLC System (Hitachi High-Tech Corp, Japan), equipped with a pump (L-2130), column oven (L-2350), autosampler (L-2200), and an Aminex HPX-87H ion-exclusion column (300 × 7.8 mm; Bio-Rad). The mobile phase was 2.2 mM H_2_SO_4_ supplied at a constant flow rate of 0.55 mL/min. The sample was injected at 10 µL and run for 25 min. The column temperature was adjusted to 18°C. The quantitative determination was carried out using an L-2490 refractive index detector and an L-2400 UV detector (210 nm).

### LC-MS analysis

Positive and negative controls to evaluate oxalurate were analyzed by LC-MS (NICEM, Republic of Korea). Liquid chromatographic analysis was performed by a Thermo Scientific Vanquish UHPLC system fitted with a Q-Exactive Quadrupole-Orbitrap Mass Spectrometer equipped with a heated electrospray ionization (H-ESI I) source. Chromatographic separation was performed on an Aminex HPX-87H ion-exclusion column (300 × 7.8 mm; Bio-Rad) at 40°C. The flow of the mobile phase of 0.1% formic acid was maintained at 0.5 mL/min. In ESI-negative ion mode, the mass spectrometer was operated as follows: spray voltage, 3.00 kV; sheath gas flow rate, 50 arbitrary units; auxiliary gas flow rate, 10 arbitrary units; ion transfer tube temperature, 320°C; and auxiliary gas heater temperature, 300°C.

The instrument was calibrated externally using the Pierce ESI-negative ion calibration solution (product number 88324, Thermo Scientific, USA) and the Pierce LTQ VELOS ESI-positive ion calibration solution (product number 88323, Thermo Scientific). Data acquisition and analysis were performed using Xcalibur 2.1 software (Thermo Scientific).

### Statistical analysis

Statistical analyses were performed using PASW Statistics 18 (SPSS, Inc.). Data were analyzed using one-way analysis of variance. *Post hoc* analyses were performed using Duncan’s multiple comparison test. Statistical significance was defined as *P* < 0.05.

## SUPPLEMENTAL MATERIAL

Fig. S1 aem.00957-24-s0001.pdf HPLC analysis of OXTCase reactions of oxamate conversion to oxalurate.

Fig. S2 aem.00957-24-s0002.pdf ^13^C NMR spectra of carbonyl peak region and ^15^N HSQC spectra of N-H peak region of the oxalurate formed from the OXTCase reaction.

Fig. S3 aem.00957-24-s0003.pdf Predicted Aligned Error (PAE) plot for AllF, AllG, and AllH using AlphaFold-multimer and AlphaFold2 through ColabFold v1.5.3.

Fig. S4 aem.00957-24-s0004.pdf Structure-based blind docking features of receptors with their ligands.

Table S1 aem.00957-24-s0005.docx Strains and plasmids used in this study.

Table S2 aem.00957-24-s0006.docx Structure-based blind docking feature of AllF, AllG, and AllH with their ligands.

Table S3 aem.00957-24-s0007.docx Presence of the genes for oxamic transcarbamylase (OXTCase) and carbamate kinase in enteric bacteria.

ASM does not own the copyrights to Supplemental Material that may be linked to, or accessed through, an article. The authors have granted ASM a non-exclusive, world-wide license to publish the Supplemental Material files. Please contact the corresponding author directly for reuse.
