# Supplementary material for: Oxamic transcarbamylase of Escherichia coli is encoded by the three genes allFGH (formerly fdrA, ylbE, and ylbF)
Source: Appl Environ Microbiol. 2024 Jun 18;90(7):e00957-24. doi: 10.1128/aem.00957-24 (PMC11326118; doi:10.1128/aem.00957-24)
Supplement: Fig. S1 — HPLC analysis of OXTCase reactions of oxamate conversion to oxalurate. [file aem.00957-24-s0001.pdf]

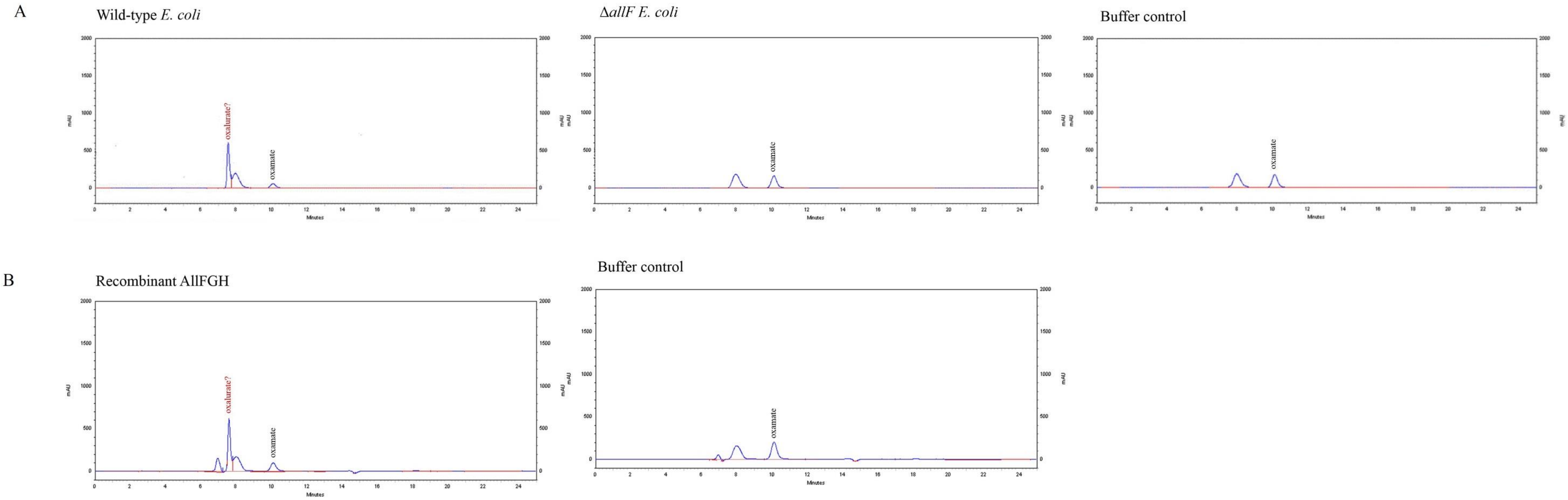

Figure S1. HPLC analysis of OXTCase reactions of oxamate conversion to oxalurate. (A) The OXTCase reaction was performed using a crude lysate of wild-type (left) and Δ*allF E. coli* strains (middle) with a buffer control (right) and (B) using the purified recombinant protein 6xHis-AllF-AllG-AllH-6xHis (left) with a buffer control (middle). Each reaction mixture contained 5 mM of oxamate, 5 mM of carbamoyl phosphate, 10 mM of $MgCl_2$, 100 mM of Tris buffer (pH 9.0), 0.1 mL lysate of wild-type *E. coli* or AllFGH, and distilled water to a volume of 1 mL. The mobile phase was 0.1% formic acid supplied at a constant flow rate of 0.55 mL/min. The sample was injected at 10 µL and run for 25 min. The column temperature was adjusted to 30°C.
