# Supplementary material for: Oxamic transcarbamylase of Escherichia coli is encoded by the three genes allFGH (formerly fdrA, ylbE, and ylbF)
Source: Appl Environ Microbiol. 2024 Jun 18;90(7):e00957-24. doi: 10.1128/aem.00957-24 (PMC11326118; doi:10.1128/aem.00957-24)
Supplement: Fig. S2 — 13C NMR spectra of carbonyl peak region and 15N HSQC spectra of N-H peak region of the oxalurate formed from the OXTCase reaction. [file aem.00957-24-s0002.pdf]

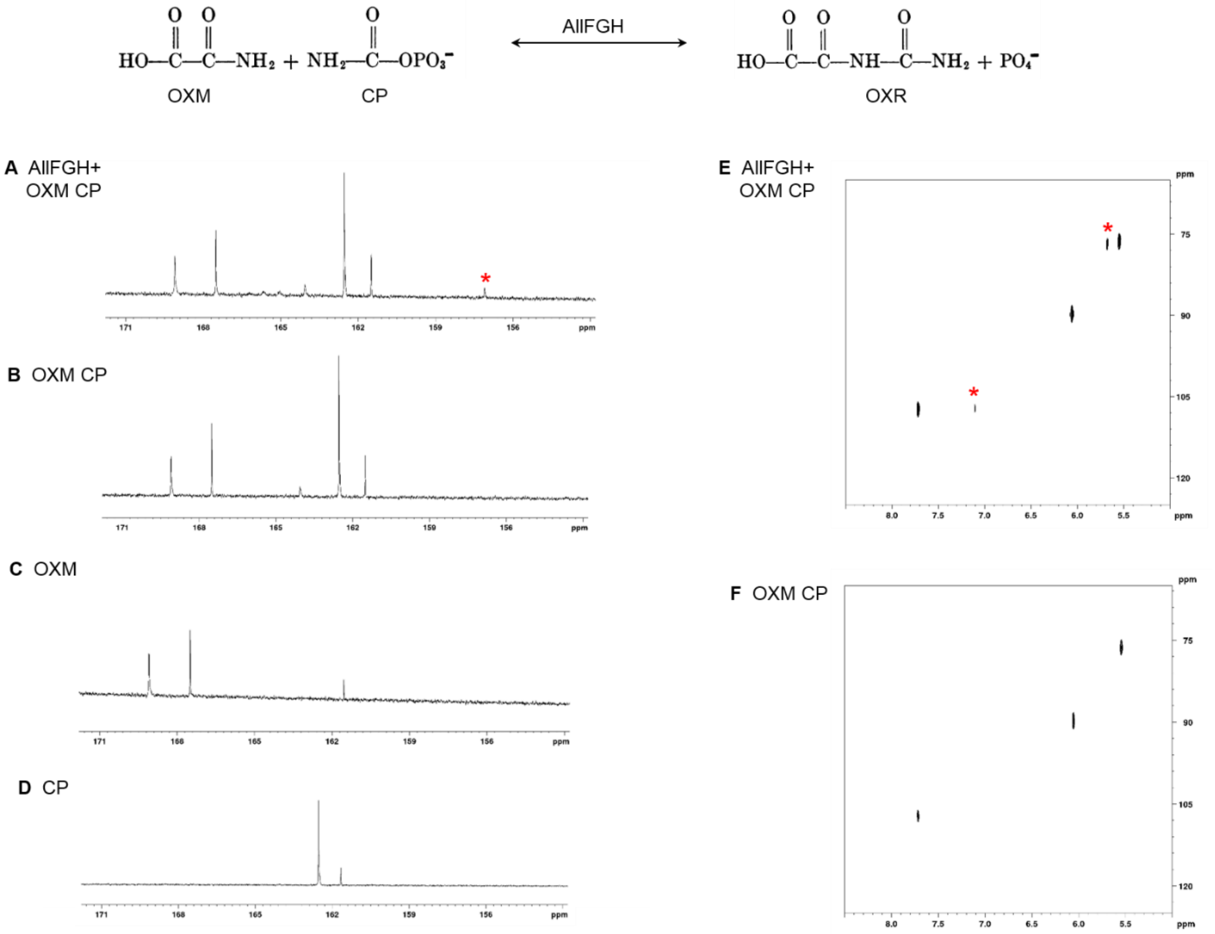

Figure S2. ^{13}C NMR spectra of carbonyl peak region (A to D) and ^{15}N HSQC spectra of N-H peak region (E and F) of the oxalurate formed from the OXTCase reaction. The OXTCase reaction of oxamate and carbamoyl phosphate (CP) to oxalurate was performed using the purified recombinant protein AllFGH (Oxalurate+) (A) or without enzyme (B) (Oxalurate-). Red asterisks show newly formed bonds in Oxalurate+. Each reaction mixture contained 20 mM of oxamate, 20 mM of carbamoyl phosphate, 5 mM of $MgCl_2$, 40 mM of Tris buffer (pH 9.0), 1 mL buffer or AllFGH, and distilled water to a volume of 10 mL. Reaction mixtures containing only each substrate, oxamate (C) or CP (D), served as standard references for the substrates. Each reaction mixture was concentrated 10-fold using a nitrogen sample concentrator. Deuterium oxide (D_2O, 10% (vol/vol), Cambridge Isotope Laboratories, Inc.) was added to each sample. ^{13}C 1D and ^{15}N HSQC NMR spectra were obtained at 25°C on a Bruker Avance III HD 800-MHz NMR spectrometer (Bruker Biospin) with a Bruker 5-mm CPTCI Z-GRD probe. Acquired spectra were phased and baseline corrected using TopSpin (version 3.6.3, Bruker BioSpin) software. The NMR analysis was performed at Korea Basic Science Institute (KBSI Metropolitan Seoul Center, Republic of Korea).
