# Supplementary material for: Oxamic transcarbamylase of Escherichia coli is encoded by the three genes allFGH (formerly fdrA, ylbE, and ylbF)
Source: Appl Environ Microbiol. 2024 Jun 18;90(7):e00957-24. doi: 10.1128/aem.00957-24 (PMC11326118; doi:10.1128/aem.00957-24)
Supplement: Fig. S3 — Predicted Aligned Error (PAE) plot for AllF, AllG, and AllH using AlphaFold-multimer and AlphaFold2 through ColabFold v1.5.3. [file aem.00957-24-s0003.pdf]

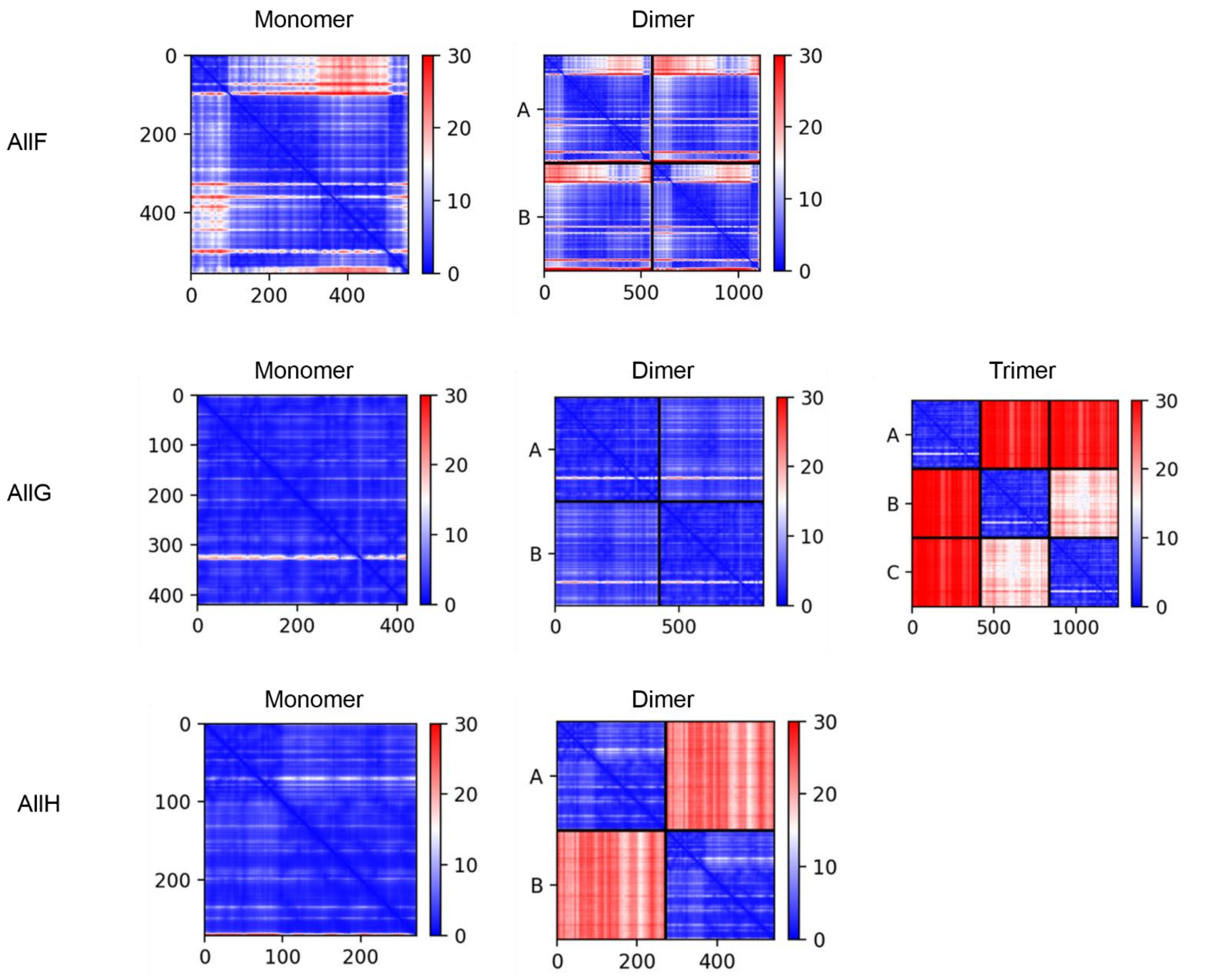

Figure S3. The Predicted Aligned Error (PAE) plot for AllF, AllG, and AllH using AlphaFold-multimer (https://colab.research.google.com/github/sokrypton/ColabFold/blob/main/AlphaFold2.ipynb?authuser=1#scrollTo=kOblAo-xetgx) and AlphaFold2 through ColabFold v1.5.3 (https://colab.research.google.com/github/sokrypton/ColabFold/blob/main/AlphaFold2.ipynb?authuser=1#scrollTo=kOblAo-xetgx).
