# Supplementary material for: Oxamic transcarbamylase of Escherichia coli is encoded by the three genes allFGH (formerly fdrA, ylbE, and ylbF)
Source: Appl Environ Microbiol. 2024 Jun 18;90(7):e00957-24. doi: 10.1128/aem.00957-24 (PMC11326118; doi:10.1128/aem.00957-24)
Supplement: Fig. S4 — Structure-based blind docking features of receptors with their ligands. [file aem.00957-24-s0004.pdf]

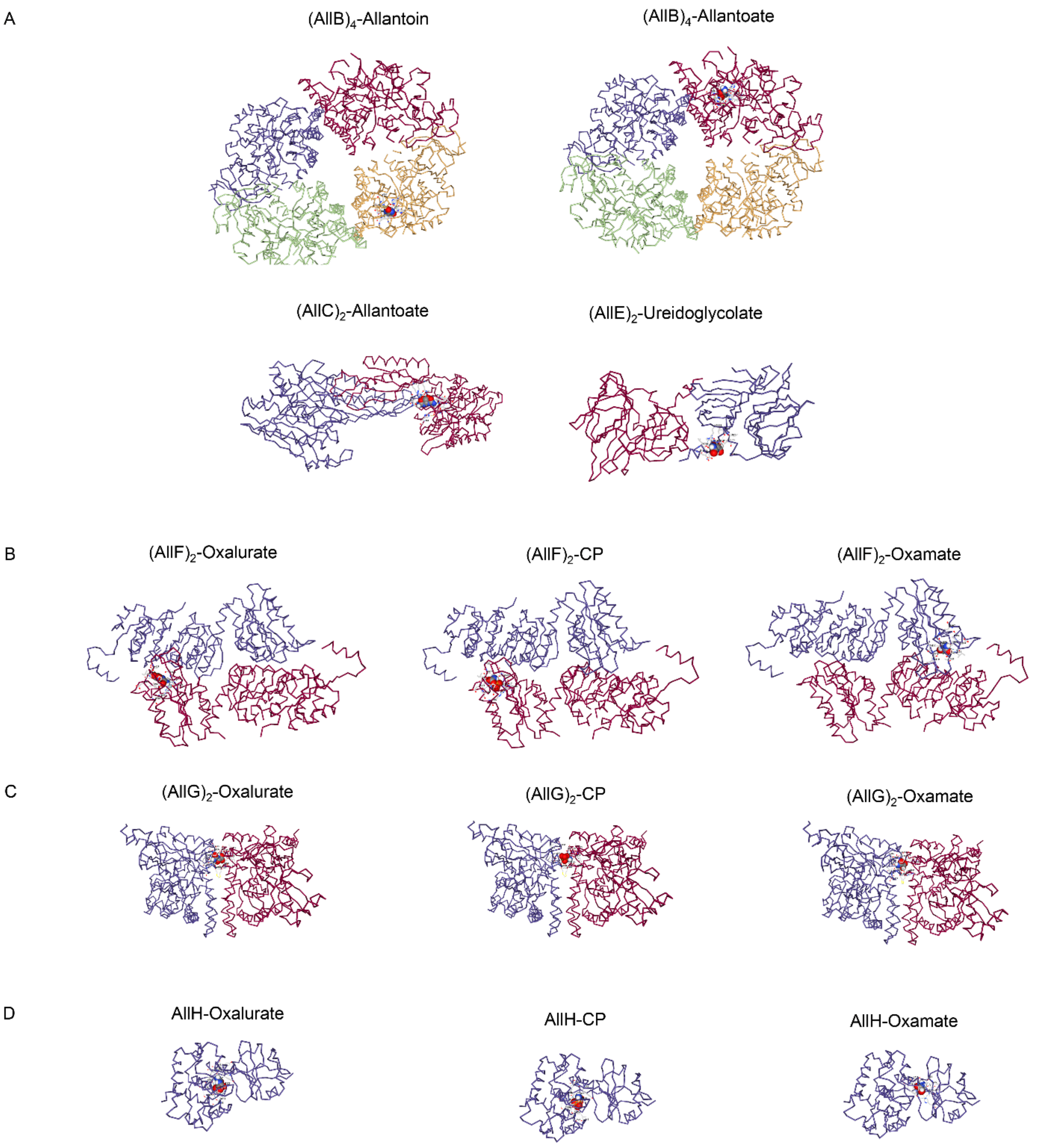

Figure S4. Structure-based blind docking features of receptors with their ligands. A. AllB, AllC, and AllE; B. $AllF_2$ (homodimer), $AllG_2$ (homodimer), and AllH (monomer); C. AllF (monomer), AllG (monomer), and AllH (monomer). The simulation was performed using the CB-DOCK2 server (https://cadd.labshare.cn/cb-dock2/php/index.php). The structures of protein (receptor) and ligand are shown as backbone and spacefill forms, respectively (data included in Table S2). The putative structures of AllF, AllG, $AllG_2$, and AllH were obtained from AlphaFold-multimer (https://colab.research.google.com/github/sokrypton/ColabFold/blob/main/AlphaFold2.ipynb?authuser=1#scrollTo=kOblAo-xetgx).
