# Supplementary material for: Oxamic transcarbamylase of Escherichia coli is encoded by the three genes allFGH (formerly fdrA, ylbE, and ylbF)
Source: Appl Environ Microbiol. 2024 Jun 18;90(7):e00957-24. doi: 10.1128/aem.00957-24 (PMC11326118; doi:10.1128/aem.00957-24)
Supplement: Table S1 — Strains and plasmids used in this study. [file aem.00957-24-s0005.docx]

Table S1. Strains and plasmids used in this study

| Strains or Plasmids | Relevant genotype | Source /References |
| --- | --- | --- |
| ***Escherichia coli* strains** |  |  |
| MG1655 | *F– λ– ilvG– rfb-50 rph-1* | (45) |
| DH5α | *fhuA2 lac(del)U169 phoA glnV44 Φ80' lacZ(del)M15 gyrA96 recA1 relA1 endA1 thi-1 hsdR17* | (46) |
| BL21(DE3) | *F– dcm ompT hsdS (rB- mB-) gal λ* | (47) |
| LMB076 | MG1655*,* but Δ*fdrA*::*cat* | (28) |
| LMB109 | MG1655*,* but Δ*ylbE*::*cat* | This study |
| LMB110 | MG1655*,* but Δ*ylbF*::*cat* | This study |
| LMB135 | MG1655*,* but Δ*fdrAylbEylbF*::*cat* | This study |
|  |  |  |
| **Plasmids** |  |  |
| pNTR-SD | Cloning vector, amp^r^ | NBRP |
| pET30b | Cloning vector, kan^r^ | Addgene |
|  |  |  |
| pNTR-SD::fdrA, | pNTR-SD with complete gene *fdrA* of MG1655, amp^r^ | (48) |
| pNTR-SD::ylbE, | pNTR-SD with complete gene *ylbE* of MG1655, amp^r^ | (48) |
| pNTR-SD::ylbF, | pNTR-SD with complete gene *ylbF* of MG1655, amp^r^ | (48) |
|  |  |  |
| pMB183 | pNTR-SD with complete genes *fdrA-ylbE-ylbF* of MG1655, amp^r^ | This study |
| pMB184 | pNTR-SD with complete genes *fdrA-ylbE* of MG1655, amp^r^ | This study |
| pMB185 | pNTR-SD with complete genes *ylbE-ylbF* of MG1655, amp^r^ | This study |
| pMB190 | pET30b with complete genes *fdrA-ylbE-ylbF* of MG1655, 6xHis-tag at C-terminus, kan^r^ | This study |
| pMB191 | pMB190 with 6xHis-tags at both C- and N-termini | This study |

References

45. Blattner FR, Plunkett G 3rd, Bloch CA, Perna NT, Burland V, Riley M, Collado-Vides J, Glasner JD, Rode CK, Mayhew GF, Gregor J, Davis NW, Kirkpatrick HA, Goeden MA, Rose DJ, Mau B, Shao Y. 1997. The complete genome sequence of *Escherichia coli* K-12. Science 277:1453–1462.

46. Taylor RG, Walker DC, McInnes RR. 1993. *E. coli* host strains significantly affect the quality of small scale plasmid DNA preparations used for sequencing. Nucleic Acids Res 21:1677–1678.

47. Jeong H, Barbe V, Lee CH, Vallenet D, Yu DS, Choi SH, Couloux A, Lee SW, Yoon SH, Cattolico L, Hur CG, Park HS, Ségurens B, Kim SC, Oh TK, Lenski RE, Studier FW, Daegelen P, Kim JF. 2009. Genome sequences of *Escherichia coli* B strains REL606 and BL21(DE3). J Mol Biol 394:644–652.

48. Saka K, Tadenuma M, Nakade S, Tanaka N, Sugawara H, Nishikawa K, Ichiyoshi N, Kitagawa M, Mori H, Ogasawara N, Nishimura A. 2005. A complete set of *Escherichia coli* open reading frames in mobile plasmids facilitating genetic studies. DNA Res 12:63–68.
