# Supplementary material for: Oxamic transcarbamylase of Escherichia coli is encoded by the three genes allFGH (formerly fdrA, ylbE, and ylbF)
Source: Appl Environ Microbiol. 2024 Jun 18;90(7):e00957-24. doi: 10.1128/aem.00957-24 (PMC11326118; doi:10.1128/aem.00957-24)
Supplement: Table S2 — Structure-based blind docking feature of AllF, AllG, and AllH with their ligands. [file aem.00957-24-s0006.docx]

Table S2. Structure-based blind docking feature of AllF, AllG and AllH with their ligands. The simulation was performed using CB-DOCK2 server (<https://cadd.labshare.cn/cb-dock2/php/index.php>). The already characterized proteins AllB (allantoin amidohydroase), AllC (allantoate amidohydrolase) and AllE (ureidoglycine aminohydroase) are used as positive reference. The putative structure of AllG, and AllH was obtained from AlphaFold-multimer (<https://colab.research.google.com/github/sokrypton/ColabFold/blob/main/AlphaFold2.ipynb?authuser=1#scrollTo=kOblAo-xetgx>).

| **Protein-Ligand** | **Vina score**  (kcal/mol) | **Center** | | | **Docking size** | | |
| --- | --- | --- | --- | --- | --- | --- | --- |
|  |  | **x** | **y** | **z** | **x** | **y** | **z** |
| (AllB)_4_-Allantoin | -6.4 | -21 | 93 | 218 | 24 | 17 | 17 |
| (AllB)_4_-Allantoate | -6.6 | -81 | 83 | 233 | 24 | 25 | 29 |
| (AllC)_2_-Allantoate | -6.3 | 64 | 37 | 25 | 26 | 31 | 23 |
| (AllE)_2_-Ureidoglycolate | -4.5 | 79 | 55 | 105 | 17 | 17 | 17 |
| (AllF)_2_-Oxalurate | -6.4 | 23 | 66 | -14 | 26 | 16 | 16 |
| (AllF)_2_-CP | -5.2 | 23 | 66 | -14 | 26 | 16 | 16 |
| (AllF)_2_-Oxamate | -4.3 | 2 | 46 | 28 | 15 | 24 | 15 |
| (AllG)_2_-Oxalurate | -6.4 | 1 | 0 | -2 | 29 | 35 | 33 |
| (AllG)_2_- CP | -5.4 | 1 | 0 | -2 | 29 | 35 | 33 |
| (AllG)_2_-Oxamate | -4.6 | 1 | 0 | -2 | 29 | 35 | 33 |
| AllH-Oxalurate | -5.6 | 2 | -7 | 1 | 25 | 25 | 16 |
| AllH- CP | -4.5 | 2 | -7 | 1 | 25 | 25 | 16 |
| AllH-Oxamate | -4.3 | 2 | -7 | 1 | 25 | 25 | 15 |
