# Supplementary material for: Oxamic transcarbamylase of Escherichia coli is encoded by the three genes allFGH (formerly fdrA, ylbE, and ylbF)
Source: Appl Environ Microbiol. 2024 Jun 18;90(7):e00957-24. doi: 10.1128/aem.00957-24 (PMC11326118; doi:10.1128/aem.00957-24)
Supplement: Table S3 — Presence of the genes for oxamic transcarbamylase (OXTCase) and carbamate kinase in enteric bacteria. [file aem.00957-24-s0007.docx]

Table S3. Presence of the genes for oxamic transcarbamylase (OXTCase) and carbamate kinase in Enteric bacteria

| Strains | Urease activity | OXTCase subunits | | | Carbamate kinase |
| --- | --- | --- | --- | --- | --- |
|  |  | AllF | AllG | AllH | AllK |
| *E. coli* MG1655 | − | ***^a^*** b0518  ***^b^*** [1129.8]  ***^c^*** [100%] | b0519  [859.4]  [100%] | b0520  [550.1]  [100%] | b0521  [598.6]  [100%] |
| *E. coli* O157:H7 Sakai | + | ECs0580  [1114.4]  [98.6%] | ECs0581  [677.9]  [78.9%] | ECs0582  [550.1]  [100%] | ECs0583  [592.0]  [98.9%] |
| *Salmonella enterica* Typhi | − | STMO0529  [935.3]  [82.8%] | STMO0530  [815.1]  [94.8%] | STMO0531  [375.5]  [68.3%] | STMO0532  [522.7]  [87.3%] |
| *Citrobacter freundii* | − | AHY12352  [960.3]  [85.0%] | AHY12353  [808.5]  [94.1%] | AHY12354  [372.9]  [67.8%] | AHY12355  [543.5]  [90.8%] |
| *Klebsiella pneumonia* | + | ***^d^*** absent | absent | absent | absent |
| *Streptococcus allantoicus* | − | ***^e^*** no information | no information | no information | no information |

−, Negative; +, Positive

***^a^*** Gene code

***^b^*** The alignment score represents the homologous protein score (bits) calculated using STRING Version 12.0.

***^c^*** The similarity represents the percentage of alignment score compared to the corresponding sequence of E. coli MG1655.

***^d^*** The corresponding gene is absent.

***^e^*** No genetic information available.
